# Association of sociodemographic and occupational stress factors with smoking behavior among healthcare professionals: The mediating role of physical exercise

**DOI:** 10.18332/tid/204007

**Published:** 2025-05-16

**Authors:** Farooq A. Chaudhary, Asma Shakoor, Muhammad A. Fareed, Osama Khattak, Mohammed S. Alqarni, Rakhi Issrani, Thani Alsharari

**Affiliations:** 1 School of Dentistry, Shaheed Zulfiqar Ali Bhutto Medical University, Islamabad, Pakistan; 2 Department of Community and Preventive Dentistry, Institute of Dentistry, Combined Military Hospital, Lahore Medical College, National University of Medical Sciences, Rawalpindi, Pakistan; 3 Clinical Sciences Department, College of Dentistry, Ajman University, Ajman, United Arab Emirates; 4 Centre of Medical and Bio-allied Health Sciences Research, Ajman University, Ajman, United Arab Emirates; 5 Department of Restorative Dentistry, College of Dentistry, Jouf University, Sakaka, Saudi Arabia; 6 Department of Oral and Maxillofacial Surgery and Diagnostic Sciences, College of Dentistry, Jouf University, Sakaka, Saudi Arabia; 7 Department of Preventive Dentistry, College of Dentistry, Jouf University, Sakaka, Saudi Arabia; 8 Department of Restorative Dental Sciences, Faculty of Dentistry, Taif University, Taif, Saudi Arabia

**Keywords:** job satisfaction, occupational stress, healthcare professionals, smoking, physical exercise

## Abstract

**INTRODUCTION:**

Smoking remains a major preventable cause of mortality, posing a significant public health challenge globally. Healthcare professionals (HCPs), despite their pivotal role in promoting health, exhibit notable smoking behaviors influenced by occupational stress and sociodemographic factors. This study investigates these relationships and examines the mediating role of physical exercise in smoking behaviors among HCPs in Pakistan.

**METHODS:**

A cross-sectional study was conducted among 302 HCPs (medical doctors and dentists) recruited using a snowball sampling technique in Pakistan from March to July 2024. Data were collected using a self-administered online questionnaire assessing sociodemographic and occupational factors, smoking status and frequency, physical exercise, sleep quality, job satisfaction, and perceived stress. Smoking behavior was analyzed as current, past, or never smokers. Mediation analysis evaluated the role of physical exercise in the association between occupational stress and smoking behavior.

**RESULTS:**

Nearly half (49.3%) of participants were current smokers, with 36.7% smoking 6–20 cigarettes daily and 60.2% of the participants experiencing moderate to high levels of perceived stress. Higher smoking prevalence was associated with gender, frequent night shifts, longer working hours, low job satisfaction, frequent insomnia, and high perceived stress (p<0.05). Regular physical exercise (52.0%) emerged as a significant protective factor against smoking, with those exercising three or more times per week being significantly less likely to smoke (OR=0.05; 95% CI: 0.03–0.09, p<0.001). Mediation analysis showed that physical exercise partially mediated the relationship between perceived stress and smoking (indirect effect = -3.67, p<0.001), with a reduced direct effect of perceived stress on smoking after controlling for exercise (B= -1.56, SE=0.22, p<0.001).

**CONCLUSIONS:**

Occupational stress, coupled with sociodemographic and work-related factors, drives smoking behaviors among Pakistani HCPs. Physical exercise serves as a protective factor, mediating the relationship between perceived stress and smoking. Workplace interventions promoting stress management, physical activity, and job satisfaction are recommended to reduce smoking rates and foster healthier behaviors among HCPs, improving public health outcomes.

## INTRODUCTION

Smoking remains one of the leading preventable causes of mortality worldwide, contributing significantly to the global burden of diseases such as cardiovascular disorders, chronic obstructive pulmonary disease, and various cancers^1^. Among healthcare professionals (HCPs), smoking behaviors present a paradox; they are often tasked with promoting healthy activities and behaviors that prevent diseases, and sometimes they engage in unhealthy practices such as smoking^2^. This contradiction raises significant public health concerns, particularly in low- and middle-income countries like Pakistan, where healthcare systems are already overburdened with resource limitations and workforce shortages^2^.

The smoking behaviors among HCPs are influenced by a complex interplay of sociodemographic and occupational factors. Previous studies have highlighted associations between smoking behavior and factors such as gender, age, marital status, workplace, working hours, and shift patterns^3,4^. For instance, younger age groups and males are often more likely to smoke, while stressful occupational environments, long working hours, and frequent night shifts exacerbate such unhealthy habits^4,5^. In Pakistan, the high prevalence of occupational stress and burden among HCPs adds another layer of complexity, potentially driving them toward maladaptive coping mechanisms such as smoking^2,6^. However, despite the significance of these associations, little research has focused on understanding the underlying pathways that link occupational stress, sociodemographic factors, and smoking behaviors in the context of HCPs in Pakistan.

Occupational stress, a psychological state that can result from workplace demands exceeding an individual’s capacity to cope, has been widely studied for its role in health-related behaviors^7-9^. Perceived stress can lead to unhealthy behaviors, including substance use, smoking, and physical inactivity. HCPs, due to their demanding roles and sensitive responsibilities, are particularly vulnerable to stress, which may, in turn, influence their smoking behaviors^7^. However, not all individuals exposed to occupational stress resort to smoking. This discrepancy suggests the presence of mediating variables that either mitigate or exacerbate the relationship between stress and smoking behavior.

Healthy behaviors, such as regular physical exercise, have been identified as potential mediators in this relationship^10^. Physical activity is known to alleviate stress, improve mental well-being, and reduce the likelihood of adopting harmful behaviors such as smoking. The mediating role of physical exercise in the stress-smoking pathway is particularly relevant in the context of HCPs, given their professional knowledge of the health benefits of exercise and their access to healthcare resources^10,11^. However, limited empirical evidence exists on how physical exercise influences the stress-smoking relationship among HCPs, especially in developing countries like Pakistan.

This study is grounded in the need to fill these research gaps, provide a comprehensive understanding of the factors influencing smoking behavior among Pakistani HCPs, and offer insights into potential interventions that can promote healthier behaviors among HCPs. Thus, the objectives of this study are: 1) to examine the association between sociodemographic and occupational stress factors with smoking behavior among HCPs in Pakistan; and 2) to assess the mediating role of physical exercise in these relationships.

## METHODS

### Study setting and participants

This cross-sectional study was carried out on medical doctors and dentists in Pakistan between March to July 2024. The ethical approval of this study was obtained from the Ethics Review Committee of the School of Dentistry, Shaheed Zulfiqar Ali Bhutto Medical University (Reference no. SOD/ERB/2024/028). Those who had a degree in Medicine or Dentistry, were actively practicing in their fields, and had internet access, were included in the study. Exclusion criteria included individuals not currently employed as healthcare professionals and those who submitted incomplete responses. The sampling pool consisted of licensed medical doctors and dentists currently practicing in Pakistan. To ensure participant eligibility, the online questionnaire began with screening questions regarding professional qualifications and active practice. Only those who identified themselves as either medical doctors or dentists were allowed to proceed to the full questionnaire. Responses from individuals who did not meet these criteria were excluded from the dataset. The initial invitation links were shared via verified channels, including the official pages and networks of teaching hospitals, professional associations, and medical/dental college faculties, to help target the appropriate population. A priori power analysis was conducted using G*Power 3.1.9.7 to determine the minimum required sample size for logistic regression analysis. Assuming a medium effect size (f^2^=0.15), alpha error probability of 0.05, power (1–β) of 0.80, and a maximum of 10 predictors in the model, the required sample size was calculated to be 150 participants. The achieved sample size of 302 participants exceeds this threshold, ensuring sufficient statistical power for the planned analyses.

### Questionnaire

Snowball sampling technique was used in recruiting the participants by sending an online link through emails, WhatsApp, Facebook, and other social media channels and, encouraging them to forward the link to their doctors and dentists contacts. Respondents who clicked on the link were directed to an information sheet that explained the purpose of the study and a consent form. Those who agreed to participate were directed to another online page that collected sociodemographic information: age (≤30, 31–51, and >50 years), gender (male, female), marital status (married, single/divorced/widowed), and profession (medical doctors, dentists). Thereafter, the participants were asked to complete a five-section questionnaire that appeared sequentially. The second part of the questionnaire collected information regarding their work: organization (government, private), work experience (<1, 1–5, 6–10, 11–15, and >16 years), hours worked (<40, 40–49, 50–59, and >60), and night shift frequency (none, 2–3, 4–5, and >6 times per month). Job satisfaction was assessed using the Job Satisfaction Survey (JSS), a 36-item questionnaire by Spector^12^; the scales include satisfaction with pay, promotional opportunities, fringe benefits, contingent rewards, supervision, co-workers, the nature of the work, communication, and working conditions. Each item uses a 6-point Likert scale that measures the degree of agreement with the statement. The overall JSS score is classified as dissatisfaction, moderate, and satisfaction, with total scores of 36–108, 109–144, and 145–216, respectively.

The third section collected information on smoking factors (smoking status: current smokers, past smokers, never smokers; and those who are current smokers answered another question regarding the number of cigarettes smoked per day). In the fourth section the information of health-related behaviors, such as physical exercise (yes, no) and sleep (frequent insomnia: yes, no), were assessed. In this study, we defined ‘regular exercise’ as exercising at least 3 times per week according to the recommendations of the National Fitness Guideline^13,14^ ‘frequent insomnia’ as having sleep disturbances (difficulty falling asleep, difficulty maintaining sleep, or waking up early) at least 3 times per week^15^. The fifth and last section assesses the perceived stress by using the Perceived Stress Scale (PSS) by Cohen^16^. Individuals click on the scores on the PSS that range from 0 to 40, with higher scores indicating higher perceived stress. Scores ranging 0–13 are considered low stress, 14–26 moderate stress, and 27–40 high stress^17,18^. Cronbach’s alpha values for the perceived stress scale and job satisfaction scale were 0.78 and 0.81, respectively, indicating good internal consistency.

### Statistical analysis

The data analysis was conducted using the Statistical Package for the Social Sciences (IBM SPSS Statistics V.26) software. Descriptive quantitative analyses were employed to analyze and summarize the survey data. To assess associations between smoking factors and qualitative variables, the chi-square test was conducted as appropriate. Given the categorical nature of the variables, to assess how perceived stress (independent variable: low, moderate, high) influences smoking behavior (dependent variable: smoking status) through mediators such as physical exercise (yes/no), binary logistic mediation analysis was conducted. Logistic regression results are reported as odds ratios (ORs) with 95% confidence intervals (CIs). The mediation analysis was carried out using three steps to evaluate different paths (a, b, c, and c’). Each path involved logistic regression models tailored to the binary nature of the dependent variables. A p<0.05 was considered statistically significant, and all statistical tests were two-tailed.

## RESULTS

A total of 302 participants were included in the study. The demographic analysis revealed that the majority of participants were aged 31–55 years (44.0%), with a higher proportion being medical doctors (60.3%), male (69.2%), and married (84.0%). Most participants worked in the government sector (65.2%) and had 1–5 years of work experience (41.1%). Additionally, 66.2% of the participants reported working between 40–59 hours per week, while only 30.5% indicated having no night shifts per month. Regarding smoking behaviors, nearly half of the participants (49.3%) were current smokers, and 36.7% reported smoking between 6–20 cigarettes per day. In terms of healthy behaviors, 52.0% engaged in physical exercise at least three times per week, and 60.6% reported no issues with insomnia. When examining perceived stress levels, 60.2% of the participants experienced moderate to high levels of perceived stress. Furthermore, only 47.4% of participants reported being satisfied with their job. These findings are summarized in Table 1.

**Table 1:** Description of characteristics of participants (N=302)

*Characteristics*	*n (%)*
**Age** (years)	
<30	85 (28.1)
31–51	133 (44.0)
>50	84 (27.8)
**Gender**	
Male	209 (69.2)
Female	93 (30.8)
**Marital status**	
Married	254 (84.1)
Single/divorced/widowed	48 (15.9)
**Profession**	
Doctor	182 (60.3)
Dentist	120 (39.7)
**Organization**	
Government	197 (65.2)
Private	105 (34.8)
**Work experience** (years)	
<1	67 (22.2)
1–7	124 (41.1)
8–15	85 (28.1)
>15	26 (8.6)
**Hours worked**	
<40	54 (17.9)
40–49	106 (35.1)
50–59	94 (31.1)
>60	48 (15.9)
**Night shift frequency** (times per month)	
None	92 (30.5)
2–3	115 (38.1)
4–5	40 (13.2)
>6	55 (18.2)
**Smoking status**	
Current smokers	149 (49.3)
Past smokers	89 (29.5)
Never smokers	64 (21.2)
**Cigarettes smoked per day**	
None	152 (50.3)
1–5	16 (5.3)
6–10	52 (17.2)
11–20	59 (19.5)
>20	23 (7.6)
**Physical exercise**	
Yes	157 (52.0)
No	145 (48.0)
**Frequent insomnia**	
Yes	119 (39.4)
No	183 (60.6)
**Perceived level of stress**	
Low	120 (39.7)
Moderate	88 (29.1)
High	94 (31.1)
**Job satisfaction**	
Dissatisfaction	99 (32.8)
Moderate	60 (19.9)
Satisfaction	143 (47.4)

The analysis of the association between sociodemographics, professional characteristics, perceived stress, and healthy behaviors with smoking behaviors revealed significant associations of gender, work experience, hours worked, night shift frequency, job satisfaction, physical exercise, insomnia, and perceived stress (p<0.05). Those with 1–7 years of experience were more likely to be current smokers, similarly, participants who worked more than 50 hours per week were more likely to smoke and those working frequent night shifts (>6 times per month) were more likely to smoke. Participants engaging in regular physical exercise were less likely to smoke, and frequent insomnia was significantly linked to higher smoking prevalence. Higher perceived stress levels correlating with increased smoking behavior and participants dissatisfied with their jobs were significantly more likely to be current smokers (Table 2).

**Table 2:** The association between sociodemographic, professional characteristics, perceived stress, healthy behaviors and smoking behaviors

	*Smoking status*			*p*	*Cigarettes smoked per day*				*p*
	*Current* *smoker* *n (%)*	*Past* *smoker* *n (%)*	*Never* *smoker* *n (%)*		*1–5* *n (%)*	*6–10* *n (%)*	*11–20* *n (%)*	*>20* *n (%)*	
**Age** (years)				0.779					0.197
<30	51 (30.0)	24 (24.0)	10 (31.1)		5 (31.3)	22 (43.1)	11 (18.6)	9 (39.1)	
31–51	72 (42.4)	46 (46.0)	15 (46.9)		7 (43.8)	17 (33.3)	31 (52.5)	9 (39.1)	
>50	47 (27.6)	30 (30.0)	7 (21.9)		4 (25.0)	12 (23.5)	17 (28.8)	5 (21.7)	
**Gender**				**0.001**					0.814
Male	120 (80.5)	63 (70.8)	26 (40.6)		12 (75.0)	41 (80.4)	47 (79.7)	20 (87.0)	
Female	29 (19.5)	26 (29.2)	38 (59.4)		4 (25.0)	10 (19.6)	12 (20.3)	3 (13.0)	
**Marital status**				0.551					0.521
Married	141 (82.9)	84 (84.0)	29 (90.6)		13 (81.3)	39 (76.5)	51 (86.4)	20 (87.0)	
Single/divorced/widowed	29 (17.1)	16 (16.0)	3 (9.4)		3 (18.8)	12 (23.5)	8 (13.6)	3 (13.0)	
**Profession**				0.910					0.946
Doctor	101 (59.4)	62 (62.0)	19 (59.4)		10 (62.5)	30 (58.8)	38 (64.4)	14 (60.9)	
Dentist	69 (40.6)	38 (38.0)	13 (40.6)		6 (37.5)	21 (41.2)	21 (35.6)	9 (39.1)	
**Organization**				0.872					0.714
Government	98 (65.8)	59 (66.3)	40 (62.5)		12 (75.0)	34 (66.7)	36 (61.0)	16 (69.6)	
Private	51 (34.2)	30 (33.7)	24 (37.5)		4 (25.0)	17 (33.3)	23 (39.0)	7 (30.4)	
**Work experience** (years)				**0.013**					**0.004**
<1	34 (22.8)	25 (28.1)	8 (12.5)		6 (37.5)	20 (39.2)	5 (8.5)	3 (13.0)	
1–7	71 (47.7)	29 (32.6)	24 (37.5)		4 (25.0)	22 (43.1)	33 (55.9)	12 (52.2)	
8–15	33 (22.1)	30 (33.7)	22 (34.4)		4 (25.0)	6 (11.8)	15 (25.4)	8 (34.8)	
>15	11 (7.4)	5 (5.6)	10 (15.6)		2 (12.5)	3 (5.9)	6 (10.2)	0	
**Hours worked**				**0.001**					**0.001**
<40	4 (2.7)	20 (22.5)	30 (46.9)		2 (12.5)	2 (3.9)	0	0	
40–49	31 (20.8)	56 (62.9)	19 (29.7)		6 (37.5)	18 (35.3)	5 (8.5)	2 (8.7)	
50–59	77 (51.7)	10 (11.2)	7 (10.9)		5 (31.3)	21 (41.2)	35 (59.3)	16 (69.6)	
>60	37 (24.8)	3 (3.4)	8 (12.5)		3 (18.8)	10 (19.6)	19 (32.2)	5 (21.7)	
**Night shift frequency** (times per month)				**0.001**					**0.007**
None	26 (17.4)	37 (41.6)	29 (45.3)		0	9 (17.6)	13 (22.0)	4 (17.4)	
2–3	44 (29.5)	49 (55.1)	22 (34.4)		8 (50.0)	16 (31.4)	10 (16.9)	10 (43.5)	
4–5	34 (22.8)	0	6 (9.4)		3 (18.8)	16 (31.4)	9 (15.3)	6 (26.1)	
>6	45 (30.2)	3 (3.4)	7 (10.9)		5 (31.3)	10 (19.6)	27 (45.8)	3 (13.0)	
**Physical exercise**				**0.001**					**0.048**
Yes	30 (20.1)	78 (87.6)	49 (76.6)		0	15 (29.4)	12 (20.3)	3 (13.0)	
No	119 (79.9)	11 (12.4)	15 (23.4)		16 (100)	36 (70.6)	47 (79.7)	20 (87.0)	
**Frequent insomnia**				**0.001**					**0.001**
Yes	98 (65.8)	3 (3.4)	18 (28.1)		14 (87.5)	22 (43.1)	42 (71.2)	20 (87.0)	
No	51 (34.2)	86 (96.6)	46 (71.9)		2 (12.5)	29 (56.9)	17 (28.8)	3 (13.0)	
**Perceived level of stress**				**0.001**					**0.043**
Low	19 (12.8)	63 (70.8)	38 (59.4)		0	11 (21.6)	6 (10.2)	2 (8.7)	
Moderate	48 (32.2)	26 (29.2)	14 (21.9)		6 (37.5)	17 (33.3)	22 (37.3)	3 (13.0)	
High	82 (55.0)	0	12 (18.8)		10 (62.5)	23 (45.1)	31 (52.5)	18 (78.3)	
**Job satisfaction**				**0.001**					**0.001**
Dissatisfaction	71 (47.7)	14 (15.7)	14 (21.9)		13 (81.3)	12 (23.5)	31 (52.5)	15 (65.2)	
Moderate	38 (25.5)	8 (9.0)	14 (21.9)		3 (18.8)	17 (33.3)	12 (20.3)	6 (26.1)	
Satisfaction	40 (26.8)	67 (75.3)	36 (56.3)		0	22 (43.1)	16 (27.1)	2 (8.7)	

Chi-squared test was used for categorical variables.

Regarding the number of cigarettes smoked per day, longer work hours and increased work experience were associated with smoking more cigarettes. Similarly, participants with frequent night shifts smoked more cigarettes per day. Those who exercised regularly were less likely to smoke heavily. Participants with high perceived stress levels or low job satisfaction smoked more cigarettes (Table 2).

The results of binary logistic regression showed (Path b) that physical exercise significantly predicted smoking status. Those who engaged in physical exercise were significantly less likely to be smokers (OR=0.05; 95% CI: 0.029–0.092, p<0.001). This indicates a strong negative association between physical activity and smoking behavior (Table 3). After controlling for physical exercise (Path c’), the direct effect of perceived stress on smoking behavior was reduced, indicating partial mediation. Specifically, the OR for perceived stress decreased from 0.16 (95% CI: 0.11–0.26) in the total effect model (Path c) to 0.20 (95% CI: 0.13–0.33) in the direct effect model (Path c’) (p<0.001), supporting the mediating role of physical exercise. When calculating the mediation effect, the indirect effect [calculated as B of Path a (1.24) × B of Path b (-2.96) = -3.67] was significant, confirming partial mediation. This shows a substantial mediation pathway from stress through physical exercise to smoking behavior. The significant reduction in the B coefficient from Path c to Path c’ (from -1.80 to -1.56) supports partial mediation (Table 3).

**Table 3:** Binary mediation analysis showing the effects of stress on smoking behavior through physical exercise

*Path*	*Factors associated with*	*Outcome*	*B*	*OR*	*95% CI*	*p*
Path a	Stress (low, moderate, high)	Physical exercise (yes, no)	1.24	3.46	2.51–4.78	0.001
Path b	Physical exercise (yes, no)	Smoking status (smoker, non-smoker)	-2.96	0.05	0.029–0.092	0.001
Path c	Stress (low, moderate, high)	Smoking status (smoker, non-smoker)	-1.80	0.16	0.112–0.243	0.001
Path c’	Stress + physical exercise	Smoking status (smoker, non-smoker)	-1.56	0.20	0.133–0.326	0.001

B: estimated coefficient.

## DISCUSSION

This study explores the relationship between sociodemographic, occupational stress factors, and smoking behavior among healthcare professionals (HCPs) in Pakistan while highlighting the mediating role of physical exercise. Our findings reinforce the growing evidence that occupational stress and its associated factors contribute significantly to smoking behavior among HCPs.

In this study, the prevalence of smoking among HCPs was 49.3% which is slightly higher compared to earlier studies reporting smoking rates among Pakistani healthcare professionals ranging from 32% to 37%^2,19^. Such high prevalence is concerning, given the role of HCPs as health promoters and the negative impact their smoking behaviors can have on public health messaging.

Occupational factors, including work experience, hours worked, and night shift frequency, were significantly associated with smoking behaviors. HCPs with 1–7 years of experience, those working more than 50 hours per week, and those with frequent night shifts (>6 times per month) were more likely to smoke. These findings suggest that early-career professionals and those with demanding work schedules may be particularly vulnerable to adopting smoking as a coping mechanism for work-related stress. This is in line with previous studies indicating that stressful occupational environments contribute to increased smoking rates among healthcare workers^20-23^.

Sociodemographic variables such as gender were associated with smoking, consistent with prior studies^24,25^. The higher smoking prevalence among male HCPs may reflect cultural and gender norms in Pakistan, where smoking is more socially acceptable for men^26^. Additionally, job dissatisfaction and frequent insomnia were significantly linked to higher smoking prevalence. This link indicating that both psychological and physiological stressors contribute to smoking behaviors, underlines the importance of workplace satisfaction as a potential buffer against stress-induced unhealthy behaviors. A large-scale study in Finland on 47000 public sector employees showed that high job strain, high effort-reward imbalance, and high job demands were associated with a higher likelihood of being a heavy smoker^27^. Addressing these factors through organizational changes and support systems may be crucial in reducing smoking rates among HCPs.

Physical exercise emerged as a significant mediator, highlighting its protective role in reducing the likelihood of smoking. Regular physical activity not only mitigates the physiological effects of perceived stress but also fosters healthier coping strategies, thereby diminishing the inclination for smoking. This finding underscores the critical need to promote physical exercise as part of workplace health initiatives for HCPs. Interestingly, participants engaging in regular physical exercise were not only less likely to smoke but also smoked fewer cigarettes if they did. This observation reinforces the inverse relationship between exercise and smoking, which has been documented in the literature, also suggesting that regular exercise can aid in smoking cessation and reduce withdrawal symptoms^28,29^. Physical activity may serve as a dual intervention, addressing both perceived stress and smoking behaviors simultaneously. The partial mediation effect observed in this study reveals that while physical exercise significantly reduces the direct impact of perceived stress on smoking behavior, other unmeasured factors may also play a role. This calls for further research to explore additional mediators, such as psychological resilience, peer support, or workplace wellness programs. The finding that frequent insomnia correlates with higher smoking prevalence adds a novel dimension to the interplay between perceived stress, sleep, and smoking. Sleep disturbances may exacerbate stress levels, creating a vicious cycle that further entrenches smoking behavior. Addressing sleep quality through workplace interventions could, therefore, yield dual benefits in reducing both perceived stress and smoking prevalence.

### Limitations

This study has some limitations. First, its cross-sectional design restricts the ability to infer causality between occupational stress, physical exercise, and smoking behavior. Longitudinal studies are needed to confirm the temporal relationships among these variables. Second, the reliance on self-reported data for smoking behaviors, physical exercise, and perceived stress levels may introduce recall and social desirability bias. Although key potential confounders were considered in the analysis, the possibility of residual confounding by unmeasured variables such as personality traits, peer influence, or environmental factors cannot be ruled out. Also, the mediation analysis relied on a product-of-coefficients approach using binary logistic regression, which assumes no unmeasured confounding between the mediator and outcome, and that the mediator is not affected by the outcome – assumptions that may not be fully testable in an observational study. Third, the use of snowball sampling may limit the generalizability of the results, as this method relies on participants recruiting others within their networks, which could introduce selection bias. Additionally, the sample was confined to healthcare professionals in Pakistan, limiting the ability to extrapolate findings to HCPs in other contexts, particularly in countries with different cultural and occupational stress dynamics. While the mediation analysis in this study was conducted using a stepwise binary logistic regression approach suitable for categorical variables, we acknowledge that more advanced techniques such as structural equation modeling (SEM) with bootstrapping or sandwich estimators could offer more robust estimates of indirect effects, especially in complex models. However, due to the binary nature of both the mediator (physical exercise) and the outcome variable (smoking status), traditional SEM was not feasible. Future studies with larger sample sizes and continuous or latent variables may consider using SEM and bootstrapping methods to further validate and expand upon these mediation pathways. Lastly, this study did not differentiate between types or intensities of physical exercise, which might have varying effects on perceived stress reduction and smoking behavior.

### Implications

From a policy perspective, the results of this study highlight the urgent need to address occupational stress and promote healthy behaviors among HCPs. Implementing stress management programs, encouraging regular physical exercise, and fostering job satisfaction may significantly reduce smoking rates in this critical workforce. Given that HCPs serve as role models for the general population, improving their health behaviors has the potential to generate ripple effects across broader public health outcomes.

## CONCLUSIONS

This study provides evidence that occupational stress, sociodemographic factors, and healthy behaviors are intricately linked to smoking behavior among healthcare professionals in Pakistan. The findings reveal that physical exercise serves as a significant mediator, reducing the impact of perceived stress on smoking while promoting healthier coping mechanisms and physical activity could serve as an effective intervention to mitigate perceived stress and reduce smoking prevalence in this population.

The implications of these findings are twofold. First, they underscore the need for targeted interventions to address occupational stress and promote physical exercise among HCPs. Second, they highlight the importance of fostering supportive work environments to mitigate perceived stress and improve job satisfaction.

## Data Availability

The data supporting this research are available from the authors on reasonable request.
